# Abiotic Stress Tolerance in Plants: Myriad Roles of Ascorbate Peroxidase

**DOI:** 10.3389/fpls.2017.00581

**Published:** 2017-04-20

**Authors:** Saurabh Pandey, Dhirendra Fartyal, Aakrati Agarwal, Tushita Shukla, Donald James, Tanushri Kaul, Yogesh K. Negi, Sandeep Arora, Malireddy K. Reddy

**Affiliations:** ^1^Plant Molecular Biology Lab, International Centre for Genetic Engineering and BiotechnologyNew Delhi, India; ^2^Department of Biotechnology, Uttarakhand Technical UniversityDehradun, India; ^3^Plant Molecular Biology Lab, Department of Botany, University of DelhiNew Delhi, India; ^4^Division of Plant Physiology, Indian Agricultural Research InstituteNew Delhi, India; ^5^Department of Basic Sciences, College of Forestry, VCSG Uttarakhand University of Horticulture and Forestry (UUHF)Ranichauri, India; ^6^Department of Molecular Biology and Genetic Engineering, G. B. Pant University of Agriculture and TechnologyPantnagar, India

**Keywords:** APX, ROS, abiotic stress, antioxidant, ASC–GSH pathway

## Abstract

One of the most significant manifestations of environmental stress in plants is the increased production of Reactive Oxygen Species (ROS). These ROS, if allowed to accumulate unchecked, can lead to cellular toxicity. A battery of antioxidant molecules is present in plants for keeping ROS levels under check and to maintain the cellular homeostasis under stress. Ascorbate peroxidase (APX) is a key antioxidant enzyme of such scavenging systems. It catalyses the conversion of H_2_O_2_ into H_2_O, employing ascorbate as an electron donor. The expression of APX is differentially regulated in response to environmental stresses and during normal plant growth and development as well. Different isoforms of APX show differential response to environmental stresses, depending upon their sub-cellular localization, and the presence of specific regulatory elements in the upstream regions of the respective genes. The present review delineates role of APX isoforms with respect to different types of abiotic stresses and its importance as a key antioxidant enzyme in maintaining cellular homeostasis.

## Introduction

Abiotic and biotic stresses are a regular feature in natural plant environment. Stress is quite unpredictable in its duration, occurrence, and intensity and thus maintaining the growth and survival is a herculean task in affected regions. Plants can perceive even a lowest environmental stress signal and reproductive stages are most sensitive to it. Reactive oxygen species or free radicals are produced as a by-product in various cellular compartments especially mitochondria and chloroplasts in association with different kinds of oxidases (Van Breusegem and Dat, 2006). These ROS are important for signaling in several growth and developmental processes and in comprehending biotic and abiotic stresses along with programmed cell death (Bailey-Serres and Mittler, 2006). But when ROS are present in excess amounts they bring about a severe damage to cellular structure and macromolecules. Scavenging systems comprising of many antioxidants and enzymes counter these ROS entities and convert them into less toxic products in the cell, sometimes even at the site of their generation. Under stress conditions, the redox homeostasis of the cell is rapidly disturbed accumulating abundant ROS (Halliwell, 2006). Antioxidant enzymes and their isoforms come into play to remove these free radicals and APX is one of the central enzymes in this system. Its role in maintaining redox balance has been seen both in normal plant life cycle and during various abiotic stress conditions, validated through various transgenic approaches. In this review, we describe the importance of APX as a key antioxidant enzyme and the myriad roles that its isoforms perform in mitigating various environmental stresses.

## Plant stress and reactive oxygen species

Plant growth and development is squarely dependent on the availability of optimal environmental conditions and nutritional factors and any deviation from these conditions constitutes stress. It induces non-specific reversible changes and responses at various functional levels which may become permanent if allowed to persist for a longer duration. Plants, being sessile, have to confront these adverse conditions, and essentially require special adaptive mechanisms to combat them. The duration and magnitude of stress determines the severity of symptoms while the physiological manifestations involve an increase in respiration, alterations in electron transport system, inhibition of photosynthesis, and reduction in biomass. The cellular responses to stress include altered cell cycle, changes in the induction of vacuolization, and cell wall organization allowing to tolerate the stress (Cramer et al., 2007; dos Reis et al., 2012). There are alterations in the anatomy, physiology, and energy consumption which increases due to a shift in cellular metabolism to maintain cellular homeostasis. Plants react to stress by either acclimatization i.e., adjusting to the new conditions and reaching a state of homeostasis or adaptations which involve permanent alterations introduced to resist stress (Chinnusamy and Zhu, 2009).

Stress is generally categorized into two primary groups: biotic and abiotic. Biotic stress is perceived when other living organisms such as weeds, microbial pathogens, and insects induce damage to the plant while abiotic stress is caused by a physical or chemical entity in the immediate environment resulting in altered growth and productivity. The growth recovery is facilitated in case of the stress being short term, of low intensity, or the plant being tolerant. But when it cannot withstand this attack, its metabolic functions are severely affected, the phenological stages are hindered and it ultimately dies. The primary abiotic stresses include water-deficit, salinity, cold, heat, and oxidative stress.

ROS are inevitable components of aerobic metabolism. Sequential reduction of molecular oxygen produces ^1^O_2_ (singlet oxygen), H_2_O_2_ (hydrogen peroxide), O_2_^.−^ (superoxide radical), and OH^.^ (hydroxyl radical) by electron transport systems in different sub-cellular compartments like cytosol, chloroplasts, mitochondria, and microbodies (Dias et al., 2014). They have critical signaling roles at lower concentrations in processes like seed germination, pollen tube growth, leaf development and senescence, root hair growth, cell elongation, embryo formation, gravitropism, self-incompatibility, and many more (Gechev et al., 2006). This is achieved with the help of redox sensitive proteins (redox protein), mobilization of calcium, phosphorylation of protein, and gene expression.

These ROS are also the resultants of alterations in cellular metabolism which are induced in response to various environmental stresses culminating in oxidative stress (Shigeoka et al., 2002). ROS activate and/or regulate the primary and secondary signaling pathways during abiotic, oxidative, wounding, or pathogen stresses by their synthesis or detoxification (Duque et al., 2013). Temporary production of ROS, also termed as “respiratory burst,” is a common feature in biotic stress occurring during early wounding or plant-pathogen interactions. Various key players in ROS signaling pathways include zinc finger proteins (Zat 12, Zat 7) and WRKY TFs. ROS are secondary messengers in ABA transduction pathway in guard cells during abiotic stress. ABA induces H_2_O_2_ to reduce water loss (Baxter et al., 2014). Salicylic acid (SA) is also reported to be a regulator of ROS during wounding (Sharma et al., 2012).

## Antioxidative defence system in plants

**Figure 1:**
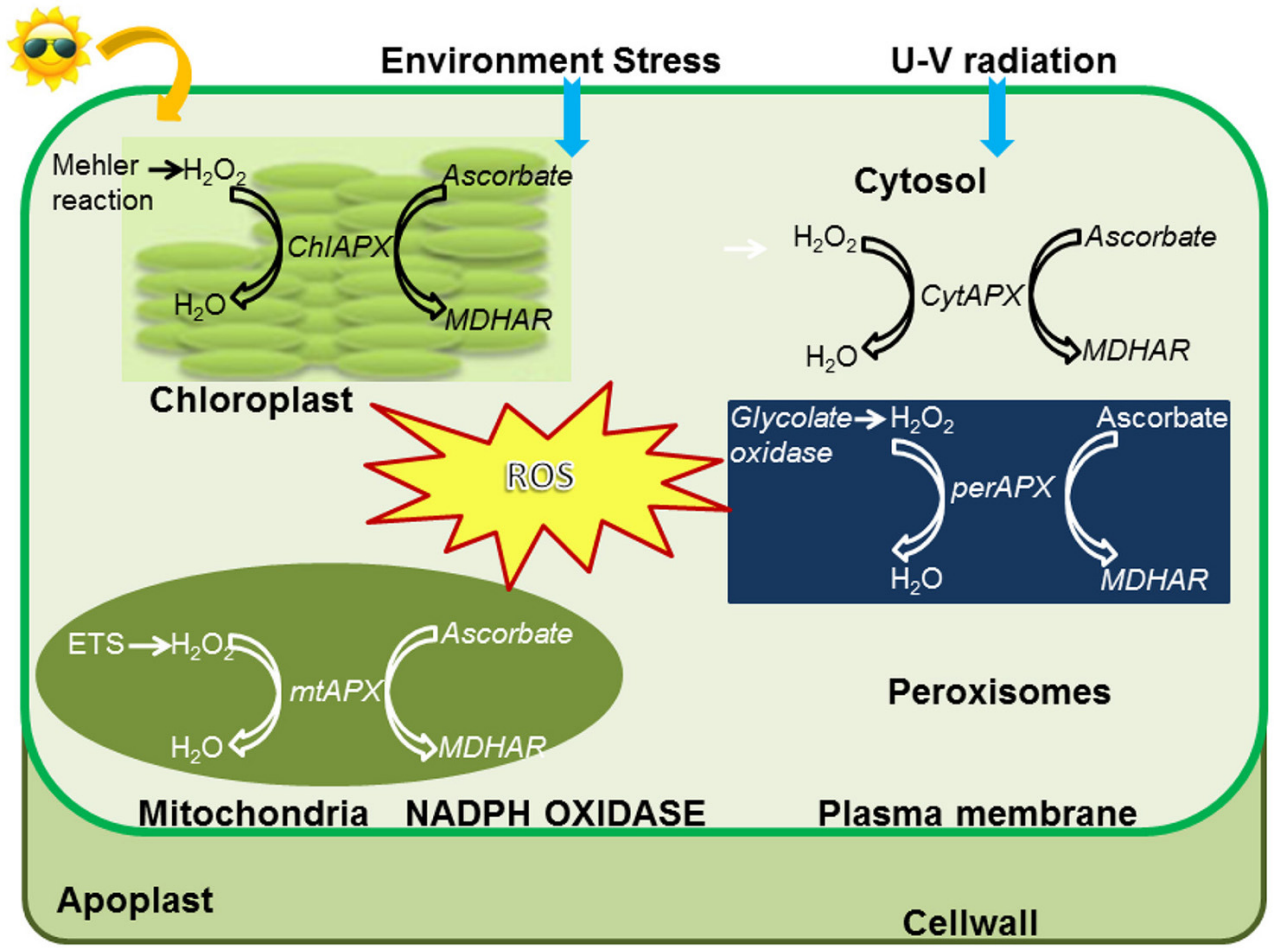
**Localization of APX enzymes and detoxification of ROS in subcellular compartments**.

The level of ROS in the cell is determined from balance between their production and scavenging by antioxidants. When ROS levels exceed a threshold required for plant metabolic processes, they become able to damage the major macromolecules of the cell i.e., proteins, lipids, and nucleic acids (Das and Roychoudhury, 2014; Kapoor, 2015). Since abiotic stresses produce abundant ROS which cause detrimental effects, its detoxification is of paramount importance to protect cellular integrity. To maintain a redox homeostasis, antioxidant defense systems are continuously activated in the plant. They comprise of two components: enzymatic and non-enzymatic. The enzymatic components (Figure 1) include superoxide dismutase (SOD), catalase (CAT), ascorbate peroxidase (APX), glutathione-S-transferase (GST), guaiacol peroxidase (GP), glutathione peroxidase (GPX), monodehydroascorbate reductase (MDHAR), dehydroascorbate reductase (DHAR), and glutathione reductase (GR). The non-enzymatic components are compounds like ascorbic acid (ASC), carotenoids, flavanoids, phenolics, reduced glutathione (GSH), and α-tocopherol which act as antioxidant buffers removing higher levels of ROS and are found in all sub-cellular compartments (Miller et al., 2010).

The stress conditions always result in an excessive production of ROS which causes a major shift in redox environment. This interferes with signaling pathways, thus, leading to the scavenging and detoxification of free radicals and other intermediate compounds through antioxidant systems (Yun et al., 2010). Excess of ${\text{O}}_{2}^{-}$ activates SOD which converts ${\text{O}}_{2}^{-}$ to H_2_O_2_ and the latter is removed by APX. Thus, these antioxidant systems eliminate excess ROS not required for basic plant processes and stabilize the internal biochemical state of the cell during various abiotic stresses, leading to acclimatization as well as tolerance (Bowler and Fluhr, 2000; Scandalios, 2005).

Ascorbate peroxidase is reported to be an efficient regulator of ROS, as it contributes maximally to hydrogen peroxide detoxification. Being present in various sub-cellular organelles, APX is also one of the most regulated enzymes (Saxena et al., 2011).

## Ascorbate peroxidase (EC 1.11.1.11)

APX belongs to the family of heme containing peroxidases that catalyse the H_2_O_2_-dependent oxidation of a wide range of organic molecules. It is present across a wide spectrum of plant kingdom and plays a crucial role in growth regulation. APX differs from other peroxidases in its dependency on ASC as the source of reducing power and becomes unstable in its absence (Shigeoka et al., 2002). There are multigenic families of APX in higher plants for e.g., *Arabidopsis* has 9 APX genes (AtAPX1-AtAPX6, sAPX, tAPX, lAPX) and *Oryza sativa* has 8 isozymes (*Os*APX1-*Os*APX8), having two APX each in cytosol, peroxisome, chloroplast and mitochondria (Table 1). Similarly, tomato has seven genes encoding different APX isozymes (Chew et al., 2003; Teixeira et al., 2004). Different isozymes of APX, which are classified on the basis of their sub-cellular localization, show different structural, and kinetic properties, including the presence of specific conserved domains and signal peptides. The thylakoidal isoform is reported to be the first one to intercept an H_2_O_2_ molecule as it is located adjacent to the acceptor of photosystem I (Huseynova et al., 2014). Broadly, on the basis of amino acid composition, five isoforms of APX have been classified in plants, *viz* cytosolic (cAPX: APX 1&2), mitochondrial (mitAPX), chloroplastic (chlAPX: stromal-APX and thylakoidal-APX), and peroxisomal/glyoxysomal (mAPX). It is interesting to note that all the isoforms of APX originate from alternative splicing, which contributes to the differential regulation of expression of various isoforms (Caverzan et al., 2012). All isoforms differ in their kinetic properties like molecular weight, optimal pH, stability, catalytic rate, and substrate affinity. As APXs are heme-dependent oxido-reductases, iron is critical for the catalytic activity and iron deficiency reduces its activity. Similar effect is observed when ascorbate concentrations are reduced, wherein the activity and stability of the enzymes is adversely affected. The cytosolic APX isoforms are more sensitive for reduction in ascorbate than chloroplastic, both stromal and thylakoid membrane-bound APX.

**Table 1:** **Localization of different APX isoforms in *Oryza sativa***.

**Gene**	**Cellular location**	**Locus I.D**.	**Chr**.	**Function**	**References**
***Oryza sativa***					
OsAPx1	Cytosolic	Os03g17690	3	Cellular response to oxidative stress, ROS salinity stress tolerance, pathogen attack	Mittler and Zilinskas, 1991; Wang et al., 2005
OsAPx2		Os07g49400	7		
OsAPx3	Peroxisomal	Os04g14680	4	Salinity and drought tolerance	Teixeira et al., 2004
OsAPx4		Os08g43560	8		
OsAPx5	Stromal	Os12g07830	12	Response to salinity stress	Yoshimura et al., 2000; Hong et al., 2007
OsAPx6		Os12g07820	12		
OsAPx7		Os04g35520	4		
OsAPx8	Thylakoid	Os02g34810	2	Involve in water- water cycle	Hong et al., 2007; Zhu et al., 2013

APXs have two important histidine residues, His_42_ and His_163_ and a K^+^ binding site which are required for APX activity. Ascorbate binds to the active site of the protein by four hydrogen bonds with lysine and arginine residues and the heme moiety, with the site of substrate binding (Cys_32_, Arg_172_, Lys_30_) being highly conserved (Chen and Asada, 1989). APX activity has been found to increase in presence of other antioxidant enzymes like superoxide dismutase and glutathione reductase, indicating a cross talk amongst various antioxidant enzymes. APX is unable to scavenge lipid hydroperoxides and is inhibited by cyanide, azide, and thiol-modifiers. The whole family of APX shows inducive responses to ABA treatment, with the cytosolic one being the most induced one (Zhang et al., 2014). The existence of multiple molecular forms of APX within the cells and organelles signifies the important role played by them in developmental processes and stress tolerance (Ishikawa et al., 1998; Shigeoka et al., 2002).

Cytosolic APX has been reported to be the most responsive isoform that is encoded by a single gene *apx1* and is also the best characterized *apx* gene. It has a heat shock responsive element in the 5′ regulatory region and an anti-peroxidative element (ARE) specifically for H_2_O_2_ scavenging. APX and its various isoforms are actively expressed under biotic stresses (pathogen attack, herbivory, physical damage) as well as under abiotic stresses (salt, drought, heat, cold, UV radiations, oxidative stress etc.) (Table 2). The extent of expression of APX directly correlates with the duration and intensity of the imposed stress, as well as the multiplicity of stresses. Further, the same kind of stress can induce differential expression of isozymes in different sub-cellular sites (Shigeoka et al., 2002).

**Table 2:** **Role of different APX isoforms in plant abiotic stress tolerance**.

**S. No**	**Gene name**	**Promoter**	**Source crop**	**Recipient crop**	**Stress**	**Outcome**	**References**
**SALT**							
1	*CytAPX*	*CaMV35S*	NA	*Solanum lycopersicum*	Salt/Chilling	*APX* activity was 10 fold higher during chilling and salt stress	Wang et al., 2005
2	*APX*	*CaMV 35S*	*Nicotiana tabacum*	*Nicotiana tabacum*	Salt/Paraquat	APX was up-regulated in multiple stresses	Lee Y. P. et al., 2007
3	*CytAPX*	*CaMV35S*	*Oryza sativa*	*Arabidopsis thaliana*	Salt	APX was up-regulated in salt stress	Lu et al., 2007
4	*PrAPX*	*CaMV35S / rd29*	*Populus sps*.	*Nicotiana tabacum*	Drought/Salt	Drought and salt tolerance during vegetative stage	Li et al., 2009
5	*StAPX*	*CaMV 35S*	*Suaeda salsa*	*Arabidopsis thaliana*	Salt	Protection against salt stress	Li et al., 2012
6	*CytAPX*	*CaMV35S/Pcambia 3301*	*Oryza sativa*	*Medicago sativa*	Salt/Drought	*APX* activity was up-regulated in salt and drought	Qian et al., 2013
7	*CytAPX*	*CaMV35S*	NA	*Prunus domestica*	Salt	Enhances the tolerance to salinity	Diaz-Vivancos et al., 2013
8	*TbAPX*	*CaMV 35S/Nos*	*Jatropha curcas*	*Nicotiana tabacum*	Salt	Enhances the tolerance to salinity	Liu et al., 2013
9	*CytAPX*	*CaMV 35S*	*Jatropha curcas*	*Arabidopsis thaliana*	Salt	*APX* activity up-regulated in salinity	Chen et al., 2015
10	*APX*	*CaMV 35S/SWPA2*	NA	*Ipomoea batatas*	Salt	*APX* up-regulated to 7.8 and 13.3 folds under salinity	Yan et al., 2016
**HEAT**							
11	*PrAPX*	*SWPA2*	*Hordeum vulgare L*.	*Arabidopsis thaliana*	Heat stress	APX up-regulated in heat stress	Shi et al., 2001
12	*ChlAPX*	*pCAMBIA2300*	NA	*Solanum tuberosum*	Heat/M-V	Up-regulated in multiple environment stresses	Tang et al., 2006
13	*StAPX*	*CaMV35S*	*Cyanidioschyzon merolae*	*Arabidopsis thaliana*	High temperature	Up-regulated during Heat stress	Hirooka et al., 2009
14	*CytAPX*	*Ubiquitin*	*Brassica campestris*	*Arabidopsis thaliana*	Heat Stress	*APX* activity up-regulated in Heat stress	Chiang et al., 2015
**COLD**							
15	*ChlAPX*	NA	*Gossypium hirsutum*	*Gossypium hirsutum*	Cold	*APX* activity up-regulated during low temperature	Kornyeyev et al., 2003
16	*TbAPX*	*35S-CaMV-LetAPX*	*LetAPX*	*Solanum lycopersicum*	Chilling stress.	Tolerance against chilling stress.	Duan et al., 2012
17	*CytAPX*	*CaMV 35S*	NA	*Brazilian arrowroot*	Cold	Improved tolerance against cold stress.	Xu et al., 2014
**DROUGHT**							
18	*APX6*	*Ubi/35S*	*Solanum melongena*	*Oryza sativa*	Water stress	Stronger resistance to flood tolerance	Chiang et al., 2015
**OXIDATIVE**							
19	*PrAPX*	NA	*Arabidopsis thaliana*	*Nicotiana tabacum*	Oxidative stress	*APX3* up-regulated in peroxisomes	Wang et al., 1999
20	*TbAPX*	*CaMV 35S*	NA	*Nicotiana tabacum*	Photo-oxidative	Up-regulated *TbAPX* activity in chloroplast	Yabuta et al., 2002
21	*APX*	*CaMV 35S*	*Nicotiana tabacum*	*Nicotiana tabacum*	M-V stress	*APX* activity highly up-regulated	Kwon et al., 2002
22	*TbAPX*	NA	*Triticum aestivum*	*Triticum aestivum*	High light stress	Role in removal of H_2_O_2_ generated during photosynthesis	Danna et al., 2003
23	*TbAPX*	*CaMV35S*	*Arabidopsis thaliana*	*Arabidopsis thaliana*	Photo-oxidation	Resistance to photo-oxidative and nitric oxide stresses	Murgia et al., 2004
24	*ChlAPX*	*SWPA2/ CaMV 35S*	NA	*Ipomoea batatas*	M-V and Chilling	APX highly up-regulated in chloroplast	Lim et al., 2007
25	*CytAPX*	NA	*Theobroma cacao*	*Moniliophthora perniciosa*	Oxidative stress	*APX* activity up-regulated in witches' broom disease	Camillo et al., 2013
**BIOTIC**							
26	*OsAPX8*	*CaMV 35S*	NA	*Oryza sativa*	Biotic	Tolerance to bacterial blight	Jiang et al., 2016
**OTHERS**							
27	*ChlAPX*	*CaMV35S*	NA	*Nicotiana tabacum*	NA	Does not provide protection against ozone	Torsethaugen et al., 1997
28	*PrAPX*	*GFP–APX3 fus*	NA	*Arabidopsis thaliana*	NA	Dispensable for *Arabidopsis thaliana* growth and development	Narendra et al., 2006

## Ascorbate-glutathione (ASC-GSH) pathway

**Figure 2:**
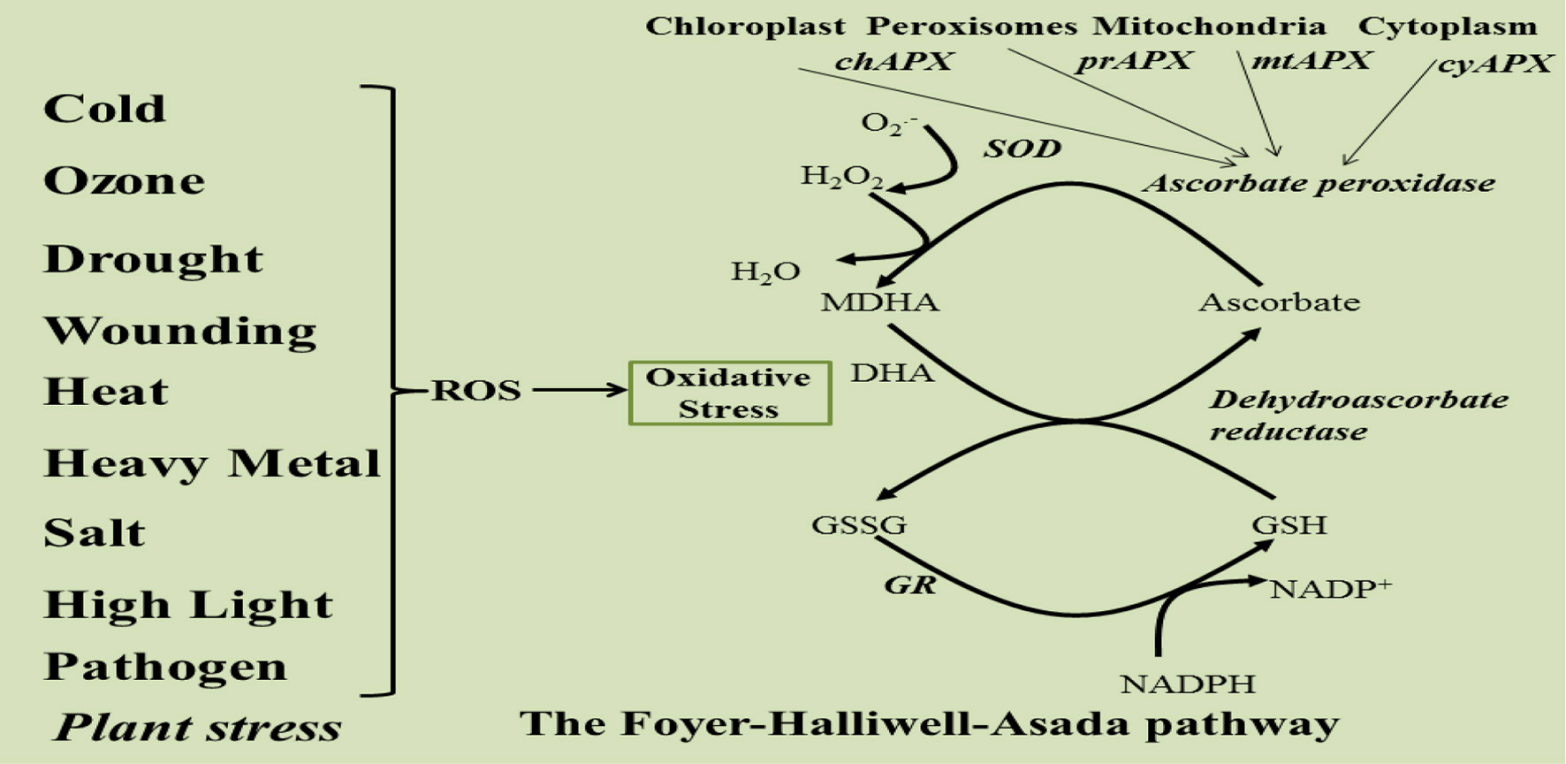
**Schematic representation of Foyer-Halliwell-Asada Pathway**.

The enzymatic and non-enzymatic antioxidant molecules work in a close coordination to give a meaningful protection from oxidative stress. Two pathways are interconnected via APX enzyme which functions as a linking molecule for maintaining the redox balance under stress. Ascorbate and glutathione have long been known to have a close association. Since both of them were assumed to have a role in detoxification, they have been studied in chloroplast samples and found to be associated with NADPH (Foyer and Halliwell, 1976, 1977). Thus, the APX-ascorbate link also serves to regulate the NADPH/NAD ratio under stress. This central scheme in which ascorbate peroxidase-ascorbate and glutathione work in tandem is known as the Ascorbate-Glutathione (ASC-GSH) pathway or Foyer-Halliwell-Asada pathway (Figure 2) that functions in both plants and animals. The importance of this pathway can be gauged from the fact that it is present in cytosol, chloroplast (stromal and thylakoid bound), mitochondria, and peroxisomes (Mittler and Zilinskas, 1991; Jimenez and Hernandez, 1997). It is known as the heart of the redox homeostasis and performs the function of a unified scavenger of ROS, although other antioxidant enzymes and components are also present in plant cells. It has been reported that detoxification of ROS through ASC-GSH pathway causes transient adjustments in the levels of most of the intermediates of this pathway (Noctor et al., 2000; Mittler et al., 2004; Noctor, 2006; Foyer and Shigeoka, 2011).

The stress generated H_2_O_2_ inactivates photosynthetic mechanism and disturbs the electron transport system and cellular respiration (Kaiser, 1976; Charles and Halliwell, 1980). Accumulation of H_2_O_2_ is highly toxic for the cell as H_2_O_2_ is the only radical species that can pass through the biological membranes and invade other sub-cellular compartments. Further, H_2_O_2_ can also lead to the production of highly reactive hydroxyl radicals in the presence of divalent cations (Figure 2). Therefore, rapid scavenging of H_2_O_2_, via ascorbate peroxidase, is extremely important for a potent antioxidant system (Asada, 1999). The possibility that ASC and GSH might function independently has been studied. Andrea Polle developed a metabolic model in 2001 as a tool to analyse the network of redox reactions in Superoxide dismutase (SOD)-ascorbate (ASC)-glutathione (GSH) cycle. The computational simulation analysis was based on previously determined concentrations of all components of the cycle, kinetic properties of antioxidative enzymes and some more others crucial parameters. The simulation results concluded a higher production rate of H_2_O_2_ in the absence of APXs without significant effects on the redox balance of ASC/DHA/ or GSH/GSSG and the coupling between ASC and GSH-related redox systems was weak (Polle, 2001).

## APX expression under various environmental conditions

APX gene expression has been reported to increase on exposure to drought, salt, cold, heat, pathogen infection, wound stress, and other biotic or abiotic stresses. However, the quantitative expression varies in different sub-cellular compartments and is also dependent on the developmental stages of the plant and the imposed stress conditions. Increase in APX activity is many times supplemented with the activity of other antioxidant enzymes that work in tandem with APX (Teixeira et al., 2006; Lee Y. P. et al., 2007).

## Direct oxidative stress

There are many biotic and abiotic factors present in the environment that are responsible for the generation of oxidative stress/ROS in plants. These factors may directly contribute to ROS production via dissipation of excess energy, reducing power, or may indirectly cause ROS production by altering the cellular metabolism. In case of methyl viologen treatment stress, chlAPX (sAPX and tAPX) is the primary target for inactivation apart from cAPX and mAPXs which show lesser sensitivity toward the chemical (Mano et al., 2001). Different plant species have differential abilities to up-regulate APX activity under stress, e.g., the tAPX usually cannot scavenge excess amounts of ROS; but when over-expressed it reduces damage under pathogen attack by reducing NO (Nitric Oxide) symptoms. Over-expression of cAPX in tobacco chloroplasts leads to increased tolerance to oxidative stress induced by paraquat as well as salinity (Dąbrowska et al., 2007). APX activity is reported to increase in *Arabidopsis* during exposure to UV-B radiations (Rao et al., 1996). cAPX transcripts were increased in germinating rice embryos upon treatment with hydroxyl-urea or amino-triazole that resulted in increased cellular H_2_O_2_ levels. Similarly, APX was up-regulated in *Arabidopsis* bundle sheath cells, upon exposure to high light intensity, due to increase in H_2_O_2_ accumulation (Karpinski et al., 1999; Morita et al., 1999). Interestingly, double mutants for APX1 and CAT1 were found to be less sensitive to oxidative stress than individual single mutants, as the former probably activated a compensatory scavenging antioxidant and defense mechanism (Rizhsky et al., 2002).

## Expression of APXs under saline conditions

Salinity stress creates ion imbalances and induces physiological drought like conditions by limiting the amount of water available to the plant. Under such conditions, APX provides salinity tolerance at different levels to the affected plants. Loss of function cAPX mutants show susceptibility to salinity induced oxidative stress, while constitutive over-expression lines show improved tolerance to 100 mM NaCl stress (Diaz-Vivancos et al., 2013). Tomato plants over expressing pea cAPX were reported to be tolerant to salinity stress (Wang et al., 2005). Over-expression of OsAPX2 shows better tolerance to salt stress as compared to OsAPX1 in transgenic *Arabidopsis* plants. However, the observed differential tolerance affect could be due to the positional effect of different transgenic lines. A cAPX from *Arabidopsis* in transgenic tobacco increased salt, drought, and PEG tolerance. Salt stress leads to lipid peroxidation and damaged membranes in sensitive plants which is accompanied by low levels of antioxidant enzymes. BY-2 cell lines of transgenic tobacco, having 50 and 75% lower cAPX activity, showed increased ROS accumulation. Ascorbate peroxidase gene expression during stress led to salt and heat tolerance with no significant changes in levels of other ROS scavenging enzymes (Gueta-Dahan et al., 1997; Badawi et al., 2004; Ishikawa et al., 2005; Lu et al., 2007). During saline conditions, pea chloroplast APXs behaved differently, with sAPX increasing and tAPX decreasing gradually while a tAPX from *Solanum lycopersicum* expressed in tobacco provided increased tolerance to salt and osmotic stress. An increased activity of chlAPX under salt stress also provides protection against ROS produced in mitochondria and/or peroxisomes. Salt stress tolerance was induced in transgenic tobacco accumulating increased ascorbate. In French bean seedlings, drought, and salt conditions up-regulated expression of gene encoding APX (Shalata et al., 2001; Mittova et al., 2003; Gómez et al., 2004; Eltelib et al., 2012; Nageshbabu and Jyothi, 2013). Over-expression of an APX from *Puccinellia tenuiflora*, a salinity tolerant wild grass in *Arabidopsis* increased its tolerance to 175mM NaCl in addition to protection from lipid peroxidation. Transcripts for a mAPX from *Hordeum vulgare* was increased under salinity conditions and another peroxisomal APX from *Populus*, transformed in tobacco, imparted salt, drought, and MV stress tolerance along with longer roots (Shi et al., 2001; Li et al., 2009). Additionally, APX deficient mutants are able to up-regulate other peroxidases to compensate for APX loss and to provide stress tolerance. This is validated by the expression of rice GPX in rice APX 1/2 mutants and other enzymes (CAT and GP) were upregulated in APX 3 knockout mutants of *Arabidopsis* showing no signs of stress. The APX expression is also affected by SA when externally applied by increasing APX and GR activity, which increases salt tolerance in mung bean (Narendra et al., 2006; Bonifacio et al., 2011; Nazar et al., 2011; Guan et al., 2015). Response of all antioxidant enzymes during salt stress in Brazilian *indica* rice was analyzed in two developmental stages and it was found that *cAPX* was up-regulated in 11 day old seedlings, while no significant APX expression was observed in 6-week old plants (Menezes-Benavente et al., 2004). This stress induces genesis of ROS, therefore, response of APX isozymes to this situation in plant developmental stages is regulated. The normal salinity tolerant rice leaf basal region showed an increase in CAT and APX transcripts under salinity stress and levels of APX8 were slightly reduced. OsAPX2 showed no alteration in expression under salinity (Yamane et al., 2010). The same reduction in response of APX8 was seen in another report where the other isoforms, APX2, and APX7 were highly expressed during salt stress. In another contrasting experiment, OsAPX8 showed high expression in a range of salt concentrations, viz., 150–300 mM in rice roots while there was a drastic decrease in transcripts of OsAPX7 at 300 mM. This variability in expression of different APX genes is due to differences in age, cultivar, plant parts, and physiological conditions of plant growth (Teixeira et al., 2004, 2006; Hong et al., 2007). Sweet potato plants differing in their sensitivity to salt stress showed differential accumulation of APX transcripts with the higher levels in tolerant genotypes. The expression of different isoforms was tissue and stress duration dependent (Lin and Pu, 2010). Salinity stress increased APX transcripts in soybean (Weisany et al., 2012). Therefore, it can be deduced that salt stress causes severe alterations in expression of antioxidant enzymes, it is spatially and temporally variable and different isoforms lead to a stable redox state in the cell.

## Expression of APXs under high light or photo-oxidative damage

High light leads to reduced efficiency of the photosynthetic apparatus and rapid ROS generation. During high light conditions, APX2 is specifically reported to have high expression while other APXs like cAPX (APX1) and tAPX transcripts increase substantially to overcome damage (Karpinski et al., 1999; Yabuta et al., 2002; Fryer et al., 2003; Pignocchi et al., 2003). cAPX and chlAPX showed differential expression in spinach leaves during high light, while the former increased, the latter decreased. A wheat mutant with deficient tAPX had lower photosynthetic efficiency in high light implying importance of this isoform (Yoshimura et al., 2000; Danna et al., 2003). Compared to wild type plants, *Arabidopsis* mutants with deficiency in either tAPX or sAPX had damaged proteins in light or MV stress with a more pronounced effect with tAPX (Maruta et al., 2010). A simultaneous mutation in two APXs of *Arabidopsis*, viz., tAPX and cAPX led to mixed results like reduced protein damage, late flowering and high anthocyanin concentrations (Miller et al., 2007b). Mutants lacking either tAPX, sAPX or both in *Arabidopsis* showed symptoms of partial chlorophyll loss in tAPX mutants, and total bleaching in seedlings for the latter two plants. Mature leaves of the same mutants were susceptible for MV and light stress (Kangasjärvi et al., 2008). *Arabidopsis* plants deficient in APX1 showed suppression in high light of transcripts of several crucial genes involved in basic plant growth and development processes. In contrast to normal conditions, high light produced induction of enzymes (Pnueli et al., 2003).

APX1 also complements chloroplastic and mitochondrial APXs in tolerating excess light and its absence leads to protein oxidation and photosynthetic failure stating its role in protection of chloroplasts (Davletova et al., 2005). But the transcript levels of chloroplastic or mitochondrial APXs do not increase unlike cAPX to tolerate stress implying their capacity to neutralize ROS even during stress. A 10-fold increase in APX expression also does not contribute to tolerance against ozone stress in tobacco chloroplasts (Torsethaugen et al., 1997). High light intensity and Mg deficiency markedly increased the expression of APX and other antioxidant enzymes in *Phaseolus vulgaris* L, while the levels in Mg-sufficient plant parts remained constant. The increase in expression was directly proportional to the light intensity (Cakmak and Marschner, 1992).

## Expression of APXs under temperature fluctuations

Extremely low or high temperature conditions negatively affect the plant physiology. Chilling stress leads to induced expression of APX in tolerant maize lines, as compared to sensitive lines (Pinhero et al., 1997). Low temperature induces higher cAPX expression in potato tubers as compared to heat stress implying its role in cold acclimation (Kawakami et al., 2002). A mAPX was induced under cold in *Arabidopsis* (Zhang et al., 1997). A tAPX over-expressed in tobacco improved tolerance to chilling and light stress while *Arabidopsis* lacking tAPX were tolerant to heat stress (Yabuta et al., 2002; Miller et al., 2007a). Homologous over-expression of a cAPX in rice was highly tolerant toward cold at booting stage due to increased activity of APX in spikelets than wild type plants (Sato et al., 2011). An inducible promoter SWPA2 working under oxidative stresses was used to induce SOD and APX gene expression in potato chloroplasts. The plants obtained were tolerant to high heat and MV stresses with a significant difference from control (Tang et al., 2006). A similar experiment in sweet potato resulted in tolerance against cold and MV stresses (Lim et al., 2007). The tomato tAPX expressed in tobacco led to tolerance against both temperature stresses and photosynthetic efficiency was maintained better in transgenic than non-transformed plants (Sun et al., 2010). In sweet potato, high heat induces cAPX in leaves while in cucumber, cAPX, mAPX, and sAPX were all up-regulated after an initial reduction (Park et al., 2004; Song et al., 2005). A cAPX has been reported to decrease immediately after heat shock treatment, negating its beneficial role in this stress (Vacca et al., 2004) but another report by Karpinski and colleague claims APX2 to be induced under heat conditions (Karpinski et al., 1999). APX1 is reported to be primarily active in case of heat and drought stresses in *Arabidopsis* cells (Koussevitzky et al., 2008). A mAPX from barley was over-expressed in *Arabidopsis* to reveal heat stress tolerance (Shi et al., 2001). Thus, different isoforms of APX and antioxidative systems in multiple sub-cellular locations can be exploited to raise environmental stress tolerant plants.

## Expression of APXs under drought conditions

APX has an important role in drought stress tolerance and recovery of plants. APX transcripts are fairly increased under drought in transgenic soybean and tobacco which over-expressed P5CS gene. In case of woody plants, APX and other ASC-GSH pathway enzymes were up-regulated after drought in *Prunus* spp. and declined during recovery phase. Glycine betaine is also reported to increase APX during drought (Sofo et al., 2005; Kausar et al., 2012; Zarei et al., 2012; Cruz et al., 2013). The cAPX (APX1) over-expression also alleviates drought symptoms and transgenic tobacco fared better than non-transgenic plants. Loss of function APX2 mutants also revealed the importance of this isoform in plant growth and development and such mutants were over sensitive to drought as compared to over-expression lines. A mAPX from *Salicornia brachiata* in over-expression lines provided increased drought tolerance compared to control plants (Zhang et al., 2013; Singh et al., 2014). Faize and colleagues revealed the importance of cAPX in drought stress tolerance in tobacco where a major beneficial effect was on membrane protection (Faize et al., 2011). The APX activity is reported to be higher in tolerant cowpea plants even in non-stress conditions. In stress, the sensitive cultivar up-regulates cAPX and mAPX, while chlAPX was up-regulated in tolerant cultivar (D'Arcy-Lameta et al., 2006). Wheat genotypes show differential APX expression under water deficit. cAPX1 was up-regulated in both genotypes, sAPX2 only in sensitive, while tAPX and cAPX2 only in the tolerant type (Sečenji et al., 2009). The tAPX was down-regulated after 15 days of stress in rice, several other isoforms were up-regulated, still some microsomal isoforms were slightly or not affected at all (Rosa et al., 2010). This represents a differential expression of APXs in different species and various stresses.

## Expression of APXs under metal toxicity

Soil contamination with heavy metal ions is a major issue hindering crop productivity. Induced expression of APXs have been observed under Cadmium and Arsenic stress in leaves of *A. thaliana, Solanum nigrum*, and *Brassica juncea* while it was reduced in *B. napus* (Smeets et al., 2008; Khan et al., 2009; Markovska et al., 2009; Nouairi et al., 2009; Pinto et al., 2009). In case of Copper stress in *Elsholtzia splendens*, there was increased expression of APX in leaves while in *Withania somnifera* it was variable according to concentration of metal ions (Peng et al., 2006; Khatun et al., 2008). In *Nicotiana tabacum* and *Typha angustifolia*, cadmium stress led to changed and unchanged expression of APX isoforms respectively while in *Typha* leaves, chromium and lead stresses did not induce any change in APX expression (Bah et al., 2011). Cadmium stress in *Zea mays* led to variable expression of APX (Ekmekçi et al., 2008). In coffee cells, lower concentrations of Cadmium induced activity of APX but higher concentrations did not cause any change after 24 h. While nickel increased APX activity with little difference between two extreme concentrations (Gomes-Junior et al., 2006a,b). Aluminum exposure also induces activity of almost all APX isoforms in rice. cAPX1/2 double mutants were normal and had increased tolerance to high concentrations (Sharma and Dubey, 2007; Rosa et al., 2010). This heavy metal increases activity of cAPX in pea, at higher concentrations and longer durations while it declines and becomes constant beyond it (Panda and Matsumoto, 2010). Iron induces activity of cAPX in de-rooted bean plants and tobacco plants with deficient cAPX were sensitive to iron (Pekker et al., 2002). Copper and cadmium increased APX activity in tall fescue plants over-expressing APX compared to control while arsenic decreased its activity in both transgenic and control (Lee S. H. et al., 2007). Lead increased APX activity in seedlings of *Eichhornia crassipes* (Malar et al., 2014). Cadmium chloride increased APX activity in both salt tolerant and sensitive rice varieties with a higher activity in the former (Roychoudhury et al., 2012; Malar et al., 2014). Similar increase in APX activity was seen in *Vigna radiata* under Cadmium Chloride stress (Roychoudhury and Ghosh, 2013). Doubled stress of salt and lead on *V. radiata* seedlings resulted in increased APX activity (Siddiqui, 2013). Therefore, we have seen an important role of APXs in protecting plants against heavy metal stress in soil from different scientific reports.

## Summary and future perspectives

From the above literature, it is amply clear that ascorbate peroxidase constitutes one of the most important component of the cellular antioxidant defense, and plays a crucial role in regulating the levels ROS in plants when exposed to a variety of environmental stresses. The fact that APX constitutes the first line of defense against ROS is signified by the fact that H_2_O_2_ at low levels is beneficial to the plant system, as it acts as a secondary messenger in initiating cellular defense pathway. The same molecule can be a cause of oxidative injury to the plant if accumulated at higher concentrations. The role of APX in maintaining redox homeostasis is supported by the presence of specific isoforms of the enzyme at different sub-cellular locations. The quantitative expression of APX regulates how the plant system adapts to different types of environmental stresses, at the cellular and molecular level by interacting with various signaling molecules. The multitude of regulatory processes and molecules present in the cell helps ascorbate peroxidase enzyme to cross talk with various other antioxidant enzymes which is essentially required for a meaningful antioxidant defense system. Further, the mutant studies indicate that APX also has a complex compensatory relationship with other enzymes of the antioxidant pathway. However, the enigma created by the presence of different isoforms of APX needs to be resolved and further studies are required to delineate the genetic regulation of *APX* gene expression, in response to different types of stresses. This will help in designing better strategies for stress tolerance in plants.

## Author contributions

SP prepared manuscript, formulated the idea, wrote the article, attached references, figures, table, and final proof reading. DF and AA formulated the idea, wrote the article, re-written and edited the manuscript, attached references, figures, table, and final proof reading. TS reproved and reorganized the manuscript. DJ helped in manuscript preparation. TK helped in guiding the article. YN helped in guiding the article. SA gave the idea of Ascorbate peroxidase (Plant APX) Review article; helped in guiding the article. MR gave the guidance for framework and final evaluation of the manuscript.

### Conflict of interest statement

The authors declare that the research was conducted in the absence of any commercial or financial relationships that could be construed as a potential conflict of interest.
